# Self- but Not Other-Dimensions of Mentalizing Moderate the Impairment Associated With Social Anxiety in Adolescents From the General Population

**DOI:** 10.3389/fpsyg.2021.721584

**Published:** 2021-11-01

**Authors:** Sergi Ballespí, Jaume Vives, Jacqueline Nonweiler, Ariadna Perez-Domingo, Neus Barrantes-Vidal

**Affiliations:** ^1^Department of Clinical and Health Psychology, Universitat Autònoma de Barcelona, Barcelona, Spain; ^2^Department of Psychobiology and Methodology of Health Sciences, Universitat Autònoma de Barcelona, Barcelona, Spain; ^3^Department of Mental Health, Fundació Sanitària Sant Pere Claver, Barcelona, Spain; ^4^Center for Biomedical Research Network on Mental Health (CIBERSAM), Instituto de Salud Carlos III, Madrid, Spain

**Keywords:** social anxiety, self-other mentalizing, social cognition, emotional knowledge, self-other functioning, impairment, resiliency, prevention

## Abstract

Mentalizing, or social cognition, refers to the brain’s higher order capacity that allows humans to be aware of one’s own and others’ mental states (e.g., emotions, feelings, intentions). While cognition in social anxiety has been broadly analyzed, there is a paucity of research regarding the role of *social* cognition. Moreover, mentalizing or social cognition research is traditionally focused on the understanding of others’ mental states, rather than self-mentalizing. Finally, most studies analyze the role of social cognition in the development or maintenance of social anxiety, yet no study to date has analyzed whether social cognition moderates functional impairment associated with it. This study analyzes whether self- and other-mentalizing moderate the relationship between social anxiety and impairment in social and self-functioning. A sample of 262 adolescents from the non-clinical population was assessed on measures of social anxiety, self- and other- mentalization, indicators of social functioning (social competence and sociometric status), and indicators of self-functioning (depression and self-esteem). Multiple linear regressions were conducted to test possible moderation effects of self-mentalizing and other-mentalizing on the relationships between social anxiety and social and self-functioning. Results revealed that other-mentalizing does not moderate social- nor self-functioning, while self-mentalizing moderates the impairment of all of them. While impairment in social functioning is buffered by one dimension of self-mentalizing (emotional clarity; *b* = 0.003, *p* = 0.043 and *b* = 0.016, *p* = 0.008 for social competence and sociometric status, respectively), impairment in self-functioning is strengthened by the other dimension (attention to emotions; *b* = −0.007, *p* = 0.008 and *b* = 0.009, *p* = 0.047 for self-esteem and depression, respectively). Probing the moderation at the 16th, 50th, and 84th percentiles revealed that the negative imbalance between dimensions (i.e., high attention and low clarity) tended to exacerbate impairment most on all indicators, while the positive imbalance (i.e., low attention and high clarity) was usually the most buffering condition. This supports that “low-flying” or implicit mentalizing provides more resilience than explicit mentalizing (i.e., high attention and high clarity). Findings suggest that the work on emotional self-awareness should be stressed in the intervention of the social anxiety spectrum conditions in order to improve prevention, functioning, and ultimately, treatments, of people impaired by symptoms of social anxiety.

## Introduction

Social anxiety is anxiety about social situations—specifically one’s performance and interactions, with a core fear of negative evaluation and judgment as being, for example, anxious, crazy, weak, intimidating or unlikeable (American Psychiatric Association, 2013). The social anxiety spectrum encompasses myriad phenomena sharing this fear (Schneier et al., 2002), and ranges from non-clinical levels of shyness or behavioral inhibition to psychopathology (i.e., social anxiety disorder, avoidant personality disorder) (Stein et al., 2004).

Once clinical, social anxiety is a disorder that typically presents first in adolescence (75% of individuals experience first onset social anxiety between ages 8 and 15), exhibits prevalence rates between 2 and 7% in the Western world, and is difficult to treat (Faravelli et al., 2000; Fehm et al., 2008; Russell and Shaw, 2009; Mayo-Wilson et al., 2014). In spite of the stress and functional impairment associated with social anxiety, only half of those affected by the disorder ever seek treatment, and those who do so typically endure 15–20 years afflicted before pursuing it (American Psychiatric Association, 2013). With the aim of avoiding this high statistic, the identification of maintenance factors and variables that moderate social anxiety could help to address social anxiety before it reaches clinical significance (e.g., earlier in its developmental course).

While the role of cognition has been broadly analyzed in social anxiety (e.g., information processing biases) (Clark and Mcmanus, 2002; Heimberg et al., 2014), the role of *social cognition* in this area has been less well-researched. Further, findings regarding social cognition are inconsistent; some studies have found difficulties with social cognition in individuals with social anxiety (Banerjee and Henderson, 2001; Pile et al., 2017), while others report the opposite finding (LaBounty et al., 2017), arguing that social anxiety leads individuals to stop and observe before interacting which provides further development of social cognition skills. Even still, some studies found no association whatsoever (Batanova and Loukas, 2011; Broeren et al., 2013; Colonnesi et al., 2017). More recently, a meta-analysis by Pearcey et al. (2020) revealed a small association between social cognition and social anxiety (*r* = −0.15). The low consistency of the findings beyond a simple low association can be attributed to the disparity in measures (experimental vs. ecological), populations (clinical vs. non-clinical; different ages), and definitions both regarding social cognition and social anxiety phenomena, used in the different studies (Plana et al., 2014; Pearcey et al., 2020).

Social cognition is defined as “cognition in which people perceive, think about, interpret, categorize, and judge their own social behaviors and those of others” (American Psychological Association, 2020). This broad definition entails several processes and dimensions, ranging from emotion recognition to attributional style or social knowledge (Plana et al., 2014), and has come to be referred to using various terms (social intelligence, Theory of Mind, mentalization, and more) in the literature interchangeably. This has promoted extensive term dispersion and overlapping concepts.

In this context, the more recent paradigm of mentalizing provides a multidimensional perspective which systematizes the field with an umbrella term, rooted in neuroscience and supported by neurobiology (Frith, 1999; Frith and Frith, 2003; Denny et al., 2012; Luyten and Fonagy, 2015), allowing researchers to gather related concepts and to reduce term-dispersion. The mentalization paradigm structures this higher order cognition in four neuroscientifically-based dimensions or polarities (Luyten et al., 2020). Thus, defined as the brain’s capacity to notice one’s own and other’s mental states (i.e., emotions, feelings, intentions, desires) (Fonagy and Luyten, 2009; Sripada et al., 2009), mentalization can be cognitive or affective, explicit (deliberate), or implicit (automatic), based on external or on internal cues, and referred to one’s own (self-mentalizing) or to others’ mental states (other-mentalizing) (Luyten et al., 2020).

While mentalizing and social cognition have been used as synonyms, the literature about social anxiety is primarily based on social cognition, and despite reference to both self- and other-behavior in social cognition, or “cognition in which people perceive, think about, interpret, categorize, and judge their own social behaviors *and those of others*” (American Psychological Association, 2020), measures of social cognition have been traditionally referred to how we know or interpret others’ mental states (i.e., others’ intentions or feelings in the social context). Consequently, there are very few studies analyzing the dimension of self-mentalizing (i.e., awareness of one’s own mental states in the social context) in social anxiety. Moreover, while most studies analyze the role of social cognition in the development and maintenance of social anxiety (Plana et al., 2014; Alvi et al., 2020), to our knowledge, no study has yet analyzed to what extent this higher order cognition moderates functional impairment in individuals with social anxiety.

Thus, the aim of this study is to analyze whether the separate dimensions *self* and *other* within mentalization moderate the impairment experienced in social anxiety, which we operationalized in the current research as difficulties with social functioning and problems with self-functioning, specifically, level of self-esteem (usually affected in social anxiety) (Farmer and Kashdan, 2014; Iancu et al., 2015) and the level of depressive symptomatology, which is often comorbid with social anxiety (Brady and Kendall, 1992; Lewinsohn et al., 1997; Brown et al., 2001; American Psychiatric Association, 2013).

As previously stated, associations between social anxiety and social cognition are primarily based on other-mentalizing (Battaglia et al., 2009). Given the inconsistent findings in this matter (see Pearcey et al., 2020) and the gap with regard to self-mentalizing, it is not clear what to expect in terms of moderation of the impairment. While it is likely to assume that how we read others’ mental states is involved in social functioning (Ballespí et al., 2021), the insight about one’s own mental states has been associated to emotional regulation (Fonagy and Target, 2002; Fonagy et al., 2005; Hill and Updegraff, 2012; Greeson et al., 2014), as well as to other processes of self- functioning (Ballespí et al., 2019). As such, we predict that other-mentalizing will more strongly moderate the association between social anxiety and social functioning (i.e., the functioning in the social world) than self-mentalizing, which will more intensely moderate impairment experienced in self-functioning. In the current study, social functioning was operationalized using measures of social competence and sociometric status, while self-functioning was operationalized using measures of self-esteem and internalizing symptoms, in this case depression.

Furthermore, two subdimensions of emotional self-awareness have been determined by factor analysis: attention to emotions and emotional clarity (Mayer and Gaschke, 1988; Salovey et al., 1995; Mayer et al., 2016). According to Salovey et al.’s (1995) model of meta-mood experience, *Attention to emotions* is defined as “the individual’s willingness to attend to feelings” or, in other words, the magnitude of one’s attention dedicated toward noticing emotions, while *Emotional clarity* refers to the ability to pinpoint and understand one’s own mood; this requires a deeper awareness or understanding of feelings (i.e., discrimination between different emotions, and perception and cognizance of them). Because self-mentalizing is a complex, higher order process, it would be incorrect to assume that simple attention to one’s own emotions equates to clear awareness or deep understanding of the emotional states.

While both the attention and clarity dimensions of Salovey et al.’s (1995) conception of emotional self-awareness are indicative of self-mentalizing, previous findings suggest that emotional clarity is more strongly associated with emotional regulation than simple attention to emotions (Extremera and Fernández-Berrocal, 2006; Salguero et al., 2012; Balluerka et al., 2013; Resurrección et al., 2014; Vine and Aldao, 2014; Eckland and Berenbaum, 2021). In fact, attention to emotions is occasionally associated with higher emotional dysregulation (Gross, 2002; Gross and John, 2003; Thompson et al., 2009, 2013; Davis and Nichols, 2016), especially when it is not combined with high emotional clarity (Ballespí et al., 2019, 2021), in which case people are more likely to become overwhelmed and face issues with emotion regulation (Gohm, 2003; Gohm et al., 2005; Kerns and Berenbaum, 2010). Accordingly, regarding the self-dimension, we predict that emotional clarity will moderate the association between social anxiety and impairment more strongly than simple attention to emotions.

Attention to emotions and emotional clarity are not separate and independent processes (Boden and Thompson, 2017), and thus beyond isolated effects, their combined effect would be interesting to study. Though research is scant regarding the pairings of attention and clarity (possibly due to the difficulty to interpret their interaction), some authors have hypothesized about their combined effect based on their individual contribution. In their review, Davis and Nichols (2016) conclude that excessive attention to emotions coupled with lack of competency to elaborate them might be deleterious for mental health. Further, Gohm et al. (2005) found fewer stress symptoms when emotional clarity and attention were uniformly high or low (i.e., balanced), but higher stress in those individuals experiencing intense emotions but lack of emotional understanding. This is consistent to the emotionally “overwhelmed” type described by Gohm (2003), which refers to a combination of high affect intensity, intermediate attention to emotions, and low clarity. Based on this literature, Boden and Thompson (2017) conclude in their meta-analysis that people who attend highly to emotions but are unable to understand them well may be more likely to become overwhelmed and to have problems with emotion regulation. Kerns and Berenbaum (2010) found, in five studies, that the overwhelmed type is associated with worse performance in different tasks. In summary, extant literature suggests that the imbalance between dimensions composed by higher attention to emotions than emotional clarity (further referred to as “negative imbalance”) is associated with worse mental health. Accordingly, we hypothesize that high values of attention to emotions combined with low values of clarity could also magnify impairments associated with social anxiety.

Regarding a possible protective effect, we also wonder which other combinations could buffer the impairment associated with social anxiety. There is no evidence about which combinations of attention and clarity lead to protective effects, and theoretical predictions are scant. On one hand, Gohm et al. (2005) suggested that both high or low levels of attention and clarity (that is, balance between dimensions) are better than an imbalance. Conversely, Salovey et al. (1995), and recently De la Barrera et al. (2021) theorized that high emotional clarity combined with moderate attention could be the best option for adjustment and regulation. We will refer here to the imbalance composed by lower attention than clarity as “positive imbalance.” However, in summary, there is evidence supporting clarity as the active ingredient, though it lacks evidence about its combined effect with different levels of attention, so it is unclear whether clarity will be more protective when combined with high or low attention to emotions.

Both social anxiety and social cognition reach high levels in adolescence (Stein and Stein, 2008), a developmental stage with high potential for early intervention or even prevention. Consequently, and in order to encompass the variability of the social anxiety spectrum, we based the study on an adolescent sample from the general population. Because both social anxiety and social cognition show differences by sex and age (Aune and Stiles, 2009) (i.e., girls are more mature in adolescence, and tend to mentalize better, but are also more prone to social anxiety) (Asher et al., 2017), all the analyses will be controlled by age and sex.

## Materials and Methods

### Participants

A sample of 262 adolescents (144 girls, 55%) between the ages of 12 and 18 years (*M* = 14.6, *SD* = 1.7) from the general population agreed to participate in the study. This sample was recruited through schools in the context of a broader project about psychopathology, personality and coping strategies in adolescence. The inclusion criterion was to be between 12 and 18 years of age, and the exclusion criterion was presence of severe mental illness such as psychosis, autism spectrum disorder, or intellectual disability. Recruitment was carried out in the schools to simplify logistics. Ten schools of similar characteristics (urbanicity, similar size, family SES, educational orientation, and methodologies, geographically close to each other) were invited to participate in the project according to their proximity to the research center. Five of these schools agreed to collaborate, and *n* = 266 families signed the informed consent to participate in the study. The principal reasons for refusal were low interest in the project, being too busy, discomfort in giving data about mental health or, in the case of some immigrant families, the inability to understand at least one of the two languages of the questionnaires (i.e., Spanish or Catalan). It was possible to obtain self-reported data from adolescents in the 98% of cases (*n* = 262), and from parents and teachers in 95% (*n* = 254) and 84% (*n* = 223) of cases, respectively. Approximately 71% of the adolescents came from families with middle socio-economic level (11.6% low; 17.7% high) and approximately 87% were Caucasian (White-European), 9% Arabic, 2% Asian, and 2% Latino.

### Instruments

*The Social Anxiety Scale for Adolescents (SAS-A)* (La Greca and Lopez, 1998) is a measure of 22 items–18 items which refer to social anxiety and four filler items. Questions include items such as “*I feel shy around people I don’t know”* and “*I’m quiet when I’m with a group of people*.” Youths self-report how much each questionnaire item is characteristic of themselves on a 5-point scale. There are three subscales, which are all structured such that a higher score indicates greater social anxiety. These three subscales are summed to comprise a total score. The Spanish adaptation (Olivares et al., 2005) of the SAS-A shows adequate internal consistency (Cronbach’s α between 0.76 and 0.91), good test-retest reliability (r ranging from 0.75 to 0.86) over a 10 day period, and evidence for convergent validity. Cronbach’s alpha in the current sample shows excellent internal consistency (α = 0.90).

*The Trait Meta-Mood Scale (TMMS)* (Salovey et al., 1995) is a short self-report measure that is designed to assess individual’s beliefs about their identification, understanding, and regulation of emotions. This self-mentalizing measure consists of 24 items which evaluates three aspects of meta-cognition—attention (*I pay a lot of attention to my feelings*), clarity (*I can sometimes say which emotions I am experiencing*), and beliefs about regulation (*I usually have an optimistic outlook, although sometimes I feel sad*). The TMMS is evaluated on a 5-point Likert scale from 1 (strongly disagree) to 5 (strongly agree). Validity evaluations show moderate internal consistency (Cronbach’s α range from 0.82 to 0.87) and good convergent and discriminant validity. The Spanish version (Fernandez-Berrocal et al., 2004), utilized in this research shows moderate-good internal consistency (Cronbach’s α from 0.86 to 0.90), and acceptable test-retest reliability (r between 0.60 and 0.83). The current sample has excellent internal consistency (α = 0.91 for the total score, α = 0.90 for attention to emotions, and α = 0.92 for emotional clarity).

*The Adolescent Mentalizing Interview (AMI)* (Ballespí and Pérez-Domingo, 2015) is a measure specifically designed to evaluate mentalizing in adolescence. It consists of two guided exercises: the first one refers to the mental states of the characters of a picture-based story and it is scored in 3 items; the second one asks about mentalizing in the relationship with two very-close others (family or close friends) (Bartholomew and Horowitz, 1991), using *demand questions* inspired in those used by Fonagy et al. (1998) in the Reflective Function Scale and scored through 4 additional items. All 7 items are scored on a 4-point Likert scale from 0 (no mentalizing) to 4 (sophisticated mentalizing). The AMI provides a total score ranged from 0 to 28, based on one dimension which explains 64% of total variance and has excellent internal consistency (Cronbach’s α = 0.90) (Ballespí and Pérez-Domingo, 2015). Concurrent validity is supported by correlations with other measures evaluating mentalization (ranging from 0.21 to 0.47) and inter-rater reliability boasts independent interview correlations from 0.79 to 0.88 (ICC = 0.91 for the total score). The internal consistency in this sample is good (α = 0.91).

*Achenbach’s System for Empirically Based Assessment (ASEBA)* is a common dimensional and empirically derived assessment of psychopathology and functioning that has good psychometric properties (Achenbach, 2021). The Spanish adaptations of the ASEBA show good internal consistency [α ranges from 0.78 to 0.97 for the Child Behavior Checklist (CBCL), for which parents are respondents] and adequate test-retest reliability (ICC from 0.85 to 0.90) (Achenbach and Rescorla, 2001). The CBCL/6-18 outlines competence in three areas—activities, social and school–along with a total competence score which comprises a sum of the three former scores (Achenbach, 2018). The social competence scale, scored by parents (*n* = 254), is used in the present study as an indicator of social functioning.

*Sociometric Index (SI)* is a brief measure designed to evaluate sociometric status in the adolescent population (Ballespí, 2013). It consists of four items scored on a Likert scale from 1 to 9, which yields a total score between 4 and 36. This study utilizes the responses of both parents and teachers combined as a multi-informant measure. Respondents were prompted regarding adolescents’ number of friends, acceptance by peers, leadership, and popularity. The SI has evidence of convergent validity with related measures, with correlations ranging between 0.2 and 0.5. Parent and teacher versions both have good to excellent internal consistency (α = 0.87 and 0.90, respectively). Principal component analysis was utilized to create this multi-informant measure. The standardized factor scores of the first component were used as a sociometric measure. Factor loadings ranged between 0.6 and 0.9, while the factor explained 55% of variability. Internal consistency of the current sample was good for parents and excellent for teachers (α = 0.83, α = 0.94, respectively).

*Rosenberg’s Self-Esteem Scale (RSES)* (Rosenberg, 1965) is a widely used measure to assess self-esteem that consists of 10 items ranked 1–5 in accordance with the degree of agreement with each statement. Items include statements such as “*I certainly feel useless at times*” and “*I am able to do things as well as most other people*.” The Spanish adaptation of the RSES has adequate psychometric properties (Martín-Albo et al., 2007). Excellent internal consistency (α = 0.90) exists in the current sample.

*Beck’s Depression Inventory (BDI-2)* (Beck et al., 1996) contains 21 items for self-evaluation, with three symptom choices that reflect the respondent’s experience over the course of 7 days. The Spanish adaptation (Sanz et al., 2003) has good psychometric properties (e.g., Cronbach’s α = 0.87). Reliability in the current sample was excellent (α = 0.90).

### Procedure

After obtaining ethical approval in accordance with the Declaration of Helsinki and evaluation by the Ethics Committee at the Universitat Autònoma de Barcelona (CEEAH 2603, Spain), participants provided written informed consent for a broader project entitled “Personality, psychopathology, and coping strategies in adolescence.” A letter distributed by the school was utilized for the purposes of informing families about objectives, relevance, and implications of the research. Next, data were recruited within the school setting. Adolescents, parents and their teachers received sealed envelopes with the questionnaires inside with an alphanumeric code that was utilized for identity encryption. Teachers were asked to complete all questionnaires for their students who agreed to participate in the research. Once the deadline for returning questionnaire forms had passed, families were contacted in the case that there were missing or out-of-range values present in their responses. The AMIs took place in private rooms at the schools. Data collection took place over the course of approximately 5 weeks in each of five schools.

### Statistical Analysis

Sample size was calculated using G^∗^Power 3.1.9 (Faul et al., 2007). For a small size effect (*f*^2^ = 0.05), α = 0.05, power (1-β) = 0.8, three exposure variables and two control variables, the sample size required was 223. Linear regressions were conducted using IBM SPSS Statistics v25.0 to test the moderation effects of self-mentalizing and mentalizing regarding others on the relationship between social anxiety and both social functioning and self-functioning variables. Age and sex have been shown to introduce differences in the variables involved; mentalization and functioning have been described by sex and age across this developmental stage (e.g., Asher et al., 2017), and thus age and sex were controlled for in all the analyses.

**FIGURE 1:**
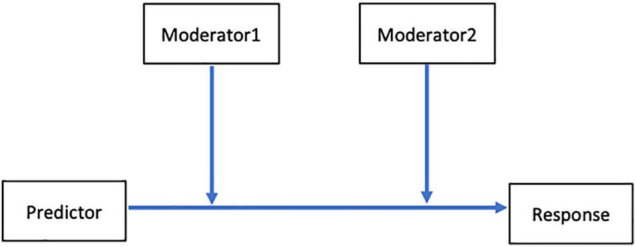
Depiction of analysis using Hayes’ (2017) PROCESS model 2.

Moderation analyses were conducted using PROCESS version 3.5, model 2 (see Figure 1; Hayes, 2017). The combined influence of both moderators was tested by probing the moderation at low, average and high values of both moderating variables, determined by 16th, 50th, and 84th percentile according to Hayes (2017). This showed the effects of social anxiety on each one of the indicators of impairment, conditioned to different values of attention to emotions and emotional clarity. This allowed us to probe how the association between social anxiety and impairment indicators changed at different levels of attention to emotions (low, average, high) combined with different levels of emotional clarity (low, average, high), and provides information about the combined influence of both moderators without the complications of a 3-way interaction. All models tested met the assumptions of normality, independent errors, homoscedasticity, and absence of multicollinearity. Results are presented as linear regression coefficients (*b*), reporting 95% confidence intervals (95% CI), and *P*-values (*p*). Statistical significance threshold was set at *p* = 0.05.

## Results

Descriptive statistics, correlations and sex differences of all variables involved are detailed in Table 1. All significant correlations were in the expected direction. Age was correlated with all mentalizing dimensions and with the indicators of self-function impairment (self-esteem and depression). There were sex differences in all variables but two: social competence and sociometric status.

**TABLE 1:** Descriptive statistics, correlations, and sex effects.

	**Descriptives**				**Correlations**							
		**Sex comparisons**										
	***M* (SD)**	**Male *M* (SD)**	**Female *M* (SD)**	**T (*p*)**	**1**	**2**	**3**	**4**	**5**	**6**	**7**	**8**
1-Social anx	45,05 (13.03)	43.75 (12.83)	46.30 (13.13)	−2.11 (0.035)	1							
2-Other M	14.45 (4.86)	13.42 (4.84)	15.35 (4.70)	−3.24 (0.001)	−0.086	1						
3- Self M-Att	23 (7.05)	21.54 (6.71)	24.22 (7.12)	−3.12 (0.002)	0.220**	0.173**	1					
4- Self M-Cla	24.81 (7.38)	27.50 (7.04)	22.58 (6.92)	5.70 (< 0.000)	−0.279**	−0.014	0.213**	1				
5- Social com.	7.66 (2.24)	7.78 (2.15)	7.56 (2.31)	0.78 (0.438)	−0.082	0.122	0.108	0.059	1			
6- Sociometric	47.35 (10.53)	47.26 (10.09)	47.43 (10.94)	−0.13 (0.900)	−0.247**	0.192**	0.122	0.109	0.481**	1		
7- Self-esteem	21.19 (5.47)	23.03 (4.92)	19.66 (5.44)	5.24 (< 0.000)	−0.565**	0.035	−0.203**	0.428**	0.129*	0.229**	1	
8- Depression	9.09 (8.28)	7.03 (7.39)	10.81 (8.62)	−3.84 (< 0.000)	0.396**	0.063	0.266**	−0.351**	−0.023	−0.108	−0.675**	1
9- Age	14.64 (1.71)	14.40 (1.66)	14.84 (1.74)	−2.08 (0.039)	0.016	0.267**	0.206**	−0.142*	−0.026	−0.040	−0.256**	0.281**

M, Mean; SD, Standard deviation; T, Student-Fisher’s t-test; p, p-value or significance degree; 1-Social anxiety; 2-Other Mentalizing; 3-Self Mentalizing-Attention; 4-Self Mentalizing Clarity; 5-Social competence; 6-Sociometric status; 7-Self-esteem (Rosenberg’s scale); 8-Depression (BDI-II). Pearson’s correlations are significant at the *0.05 level or at the **0.01 level (2-tailed). Bold indicates that the value met statistical significance.

Models with both self-mentalizing moderators (i.e., attention and clarity) and the other-mentalizing moderator were first tested for the four response variables–social competence, sociometric status, self-esteem, and depression. Other-mentalizing showed no statistically significant moderator effect on social competence (*b* = 0.002; *p* = 0.245; 95% CI: −0.002 to 0.007), sociometric status (*b* = −0.018; *p* = 0.055; 95% CI: −0.036 to 0.001), self-esteem (*b* = −0.001; *p* = 0.848; 95% CI: −0.008 to 0.007) nor depression (*b* = 0.005; *p* = 0.397; 95% CI: −0.007 to 0.018) response variables, so the moderation and the conditional effect of other-mentalizing were removed from all models.

**FIGURE 2:**
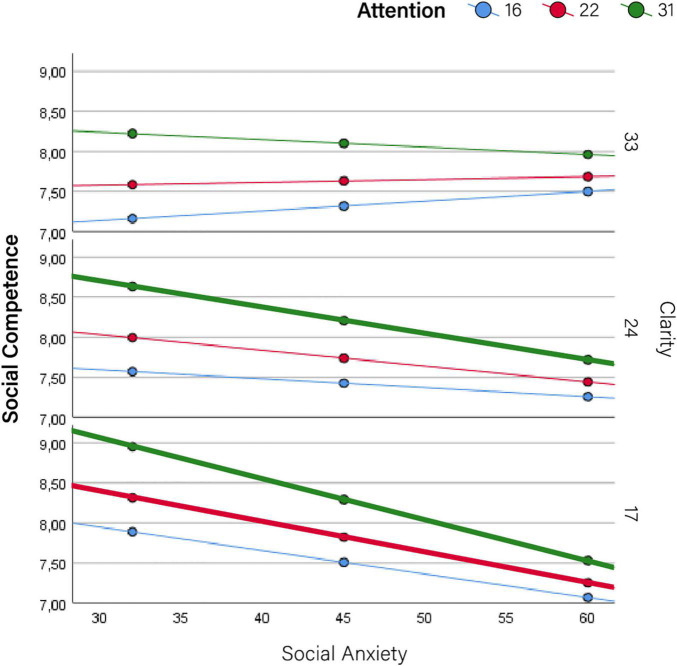
Moderator effects of self mentalizing dimensions on social competence. Conditional effects of social anxiety on social competence at different levels of attention to emotions and emotional clarity.

**FIGURE 3:**
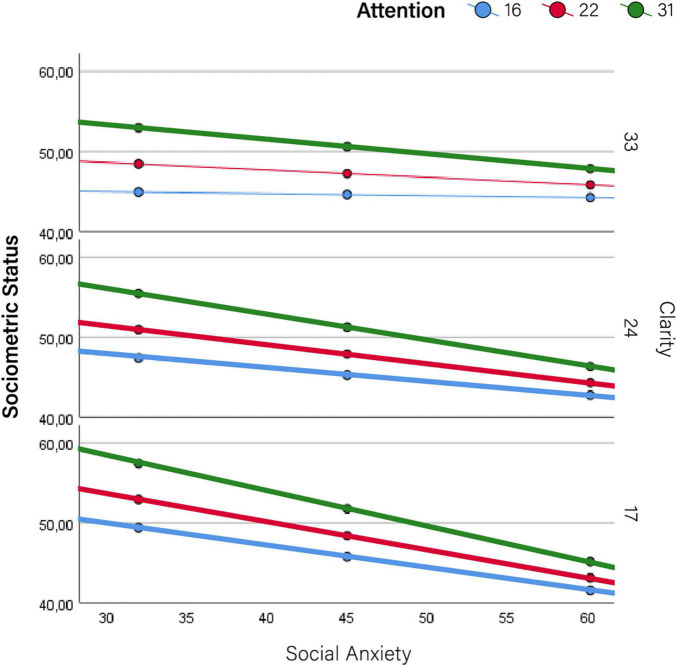
Moderator effects of self mentalizing dimensions on sociometric status. Conditional effects of social anxiety on sociometric status at different levels of attention to emotions and emotional clarity.

**FIGURE 4:**
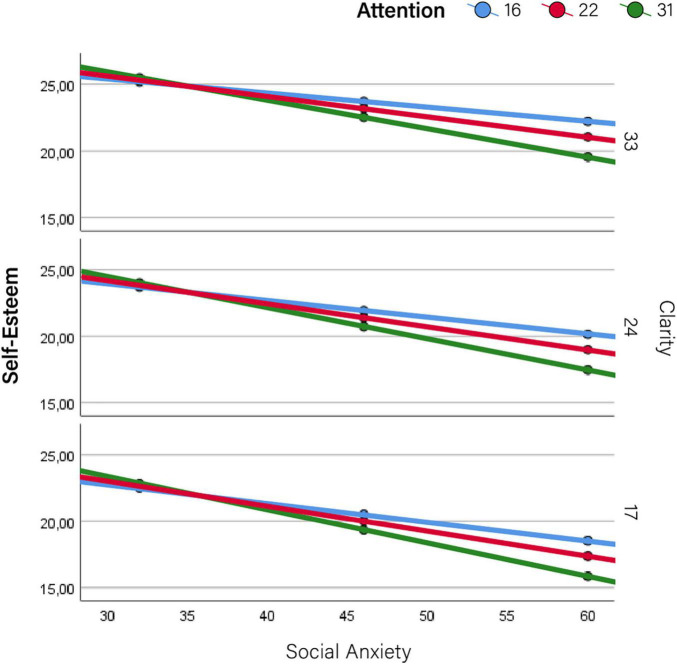
Moderator effects of self mentalizing dimensions on self-esteem. Conditional effects of social anxiety on self-esteem at different levels of attention to emotions and emotional clarity.

**FIGURE 5:**
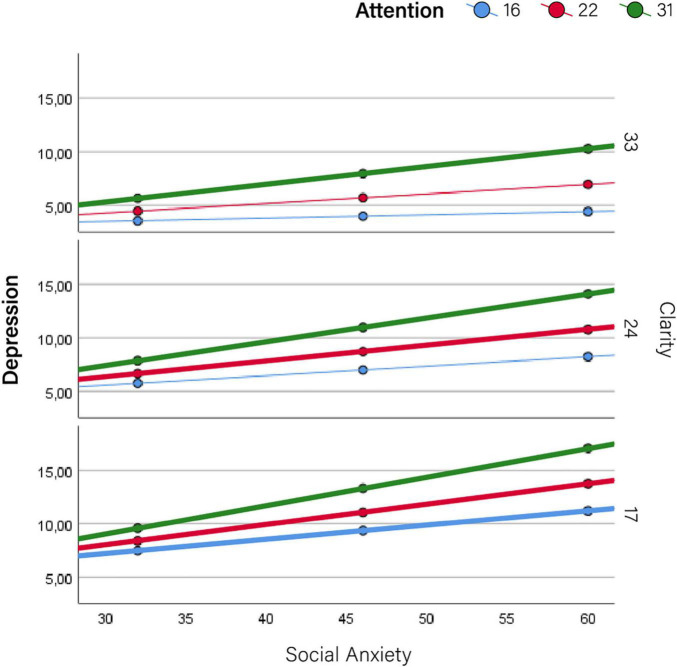
Moderator effects of self mentalizing dimensions on depression. Conditional effects of social anxiety on depression at different levels of attention to emotions and emotional clarity.

Therefore, results are primarily devoted to the moderator effects of self-mentalizing variables on the relationship between social anxiety and indicators of impairment. These results are summarized in Tables 2, 3 (conditional effects on social- and self-function, respectively), which show the effect sizes (*b*) of social anxiety (conditional to moderators being set at their mean values) along with those of self-mentalizing moderators (attention to emotions and emotional clarity). These results are graphically depicted in Figures 2–5, which also display the expected values of the response variables (social competence, sociometric status, self-esteem, and depression) for low (16th percentile) average (50th percentile), and high (84th percentile) values of social anxiety, attention to emotions and emotional clarity. The values of these three percentiles allowed us to explore the moderation across the range of measurement without overcrowding the graphics. In line with prevalence rates and previous research, the results were controlled for sex and age. The combination of values of attention and clarity where the association between social anxiety and the response variable is statically significant are highlighted in the Figures 2–5.

**TABLE 2:** Social anxiety (conditional) effects and self-mentalizing moderation effects on social function.

	***Social functioning***					
	**Social competence (*n* = 254)**			**Sociometric status (*n* = 223)**		
	***b* (*p*)**	**95% CI**	***R* ^2^**	***b* (*p*)**	**95% CI**	***R* ^2^**
*Social anxiety*	−0.019 (0.097)	−0.042 to 0.004		−**0.231 (<0.000)**	−**0.335 to -0.127**	
M: SA × Attention	−0.001 (0.326)	−0.004 to 0.001	0.004	−0.009 (0.145)	−0.023 to 0.003	0.009
M: SA × Clarity	**0.003 (0.043)**	**0.0001–0.005**	0.016	**0.016 (0.008)**	**0.004–0.027**	**0.028**

*Results are adjusted for age and sex in all cases. R^2^, R^2^ change; M, Moderation; SA, Social Anxiety; Social competence model R^2^ = 0.046; Sociometric status model R^2^ = 0.146. Bold indicates that the value met statistical significance.*

**TABLE 3:** Social anxiety (conditional) effects and self-mentalizing moderation effects on self-function.

	**Self-function**					
	**Self-esteem (*n* = 262)**			**Depression (*n* = 262)**		
	***b* (*p*)**	**95% CI**	***R* ^2^**	***b* (*p*)**	**95% CI**	***R* ^2^**
Social anxiety	−**0.174 (<0.000)**	−**0.214 to**−**0.134**		**0.146 (<0.000)**	**0.079–0.214**	
M: SA × Attention	−**0.007 (0.008)**	−**0.012 to**−**0.002**	0.015	**0.009 (0.047)**	**0.0001–0.018**	0.011
M: SA × Clarity	0.002 (0.32)	−0.002 to 0.007	0.002	−0.006 (0.085)	−0.014 to 0.001	0.007

*Results are adjusted for age and sex in all cases. R^2^, R^2^ change; M, Moderation; SA, Social Anxiety; Self-esteem model R^2^ = 0.471; Depression model R^2^ = 0.333. Bold indicates that the value met statistical significance.*

Overall, Tables 2, 3 show a negative association between social anxiety and both indicators of social functioning (i.e., social competence and sociometric status, though it was only significant for sociometric status) as well as self-esteem, while there was a positive association between social anxiety and depression. While all the interaction terms certainly had low values of *R*^2^, Tables 2, 3 demonstrate that the association between social anxiety and functioning was moderated by facets of self-awareness. Regarding social functioning (Table 2), the negative association between social anxiety and both social competence and sociometric status was moderated by emotional clarity, such that the higher emotional clarity, the more dampened association is between social anxiety and social impairment according to both social functioning variables. As such, emotional clarity attenuated the association between social anxiety symptoms and outcome measures. Regarding self-functioning (Table 3), the relationships between social anxiety and both self-esteem and depression were moderated by attention to emotions, such that more attention to emotions strengthened the associations. Therefore, attention to emotion increased the impairment according to both indicators of self-function (i.e., the more self-esteem decreases, and the more depression increases).

More detailed results show the conditional effects of social anxiety on the different outcomes at different levels of the two self-mentalizing variables (Table 4). This information is summarized graphically in Figures 2–5. Overall, this table shows that attention to emotions tended to exacerbate impairments while emotional clarity attenuated them. When evaluating the combination of values of both moderating factors (i.e., how the association between social anxiety and impairment changed at different combinations of low/average/high values of attention and clarity), impairments were lower when clarity was higher and emotional attention was lower. Accordingly, the lowest impairment appeared when attention was low and clarity was high–that is, when there was a positive imbalance between these dimensions–while the highest impairment in all cases (i.e., the most intense association between social anxiety and each indicator) appeared when attention was high and clarity was low, that is, when there was a negative imbalance. Detailed results for each outcome are provided below.

**TABLE 4:** Conditional effects of SA at different levels of attention to emotions and emotional clarity.

	**Emotional clarity**	**Attention to emotions**	***b***	***P***	**95% CI lower–upper**
Social competence		Low	0.012	0.529	−0.026 to 0.050
	High	Average	0.004	0.819	−0.027 to 0.034
		High	−0.009	0.610	−0.045 to 0.027
		Low	−0.011	0.474	−0.042 to 0.020
	Average	Average	−0.020	0.09	−0.043 to 0.003
		High	−0.033*	0.043	−0.064 to −0.001
		Low	−0.029	0.105	−0.065 to 0.006
	Low	Average	−0.038*	0.014	−0.068 to −0.008
		High	−0.051*	0.01	−0.089 to −0.012
Sociometric status		Low	−0.024	0.798	−0.210 to 0.161
	High	Average	−0.093	0.207	−0.237 to 0.051
		High	−0.182*	0.038	−0.353 to −0.010
		Low	−0.166*	0.033	−0.317 to −0.014
	Average	Average	−0.234*	<0.000	−0.340 to −0.129
		High	0.323*	<0.000	−0.468 to −0.178
		Low	−0.278*	<0.002	−0.450 to −0.106
	Low	Average	−0.347*	<0.000	−0.484 to −0.210
		High	−0.436*	<0.000	−0.609 to −0.262
Self esteem		Low	−0.106*	0.002	−0.174 to −0.038
	High	Average	−0.152*	<0.000	−0.206 to −0.098
		High	−0.212*	<0.000	−0.277 to −0.148
		Low	−0.126*	<0.000	−0.182 to −0.071
	Average	Average	−0.172*	<0.000	−0.213 to −0.132
		High	−0.232*	<0.000	−0.289 to −0.176
		Low	−0.142*	<0.000	−0.206 to −0.078
	Low	Average	−0.188*	<0.000	−0.241 to −0.134
		High	−0.248*	<0.000	−0.316 to −0.180
Depression		Low	0.031	0.592	−0.083 to 0.146
	High	Average	0.089	0.056	−0.002 to 0.180
		High	0.165*	0.003	0.056–0.274
		Low	0.089	0.063	−0.005 to 0.183
	Average	Average	0.147*	<0.000	0.078 to 0.215
		High	0.222*	<0.000	0.127 to0.318
		Low	0.134*	0.016	0.025 to0.242
	Low	Average	0.192*	<0.000	0.101 to0.282
		High	0.267*	<0.000	0.152 to0.382

Low, 16th percentile; Average, 50th percentile; High, 84th percentile according to Hayes (2017). *Denotes statistically significant effects (p < 0.05).

### Social Functioning: Social Competence

As Table 2 shows, social anxiety was associated with all response variables except social competence, but the fact that emotional clarity still had a significant moderating role (*b* = 0.003; *p* = 0.043; 95% CI: 0.0001–0.005) implies that social anxiety has an influential effect on social competence for certain values of emotional clarity and attention to emotions. Moreover, when emotional clarity was low and attention to emotions was high or average, there was a negative association between social anxiety and social competence (*b* = −0.051, *p* = 0.01; *b* = −0.038, *p* = 0.014, respectively) (Table 4 and Figure 2). This indicates that with low emotional clarity, social competence becomes impaired unless attention to emotions is also low. Further, when attention to emotions was high and emotional clarity is average (i.e., not high), social competence was also impaired (*b* = −0.33, *p* = 0.043, respectively) (Table 4, see also Figure 2 where highlighted lines indicate the significant moderating effects).

### Social Functioning: Sociometric Status

Social anxiety was negatively associated with sociometric status (*b* = −0.231; *p* < 0.000; 95% CI: -0.335 to -0.127) and this association was moderated by emotional clarity (*b* = 0.016; *p* = 0.008; 95% CI: 0.004–0.027) (Table 2). The positive value of the moderator indicates that the association between social anxiety and sociometric status became less negative as emotional clarity increased; in other words, the slopes that represent the relationship between social anxiety and sociometric status were flattened as clarity improved. This can be clearly seen in Figure 3, which shows the effect of each moderator at different values of the other moderator. The effect of social anxiety on sociometric status is significant in all possible combinations, but was attenuated with increased emotional clarity and decreased emotional attention (see the specific values in Table 4).

### Self-Functioning: Self-Esteem

Table 3 shows that social anxiety was negatively associated with self-esteem (*b* = −0.174; *p* < 0.000; 95% CI: −0.214 to −0.134), but in this case the moderation effect came from attention to emotions (*b* = −0.007; *p* = 0.008; 95% CI: −0,012 to −0.002). The negative value of the moderation effect entails that the more attention was paid to emotions, the lower expected self-esteem as social anxiety increased (Figure 4).

### Self-Functioning: Depression

Finally, there was a statistically significant association between social anxiety and depression, but in this case a positive one (i.e., depression increased as social anxiety does) (*b* = 0.146; *p* < 0.000; 95% CI: 0.079–0.214), and this was strengthened by attention to emotions (*b* = 0.009; *p* = 0.047; 95% CI: 0.0001–0.018) (Table 4 for values, graphically depicted in Figure 5).

## Discussion

The aim of this study was to analyze whether the self- and other-dimensions of mentalizing moderate the association between social anxiety and different indicators of impairment and well-being. According to previous research, it was predicted that other-mentalizing would be more implicated in moderating the association between social anxiety and social functioning, while self-mentalizing would be more strongly involved in moderating self-functioning impairments. Curiously, no moderation was found for other-mentalizing, though self-mentalizing subdomains moderated the association between social anxiety and the indicators of social functioning (social competence and sociometric status), and self-functioning (self-esteem and depression).

### Other-Mentalizing: Lack of Moderation

The present findings are intriguing for two reasons. First, the psychopathological core of social anxiety is the fear of scrutiny and negative evaluation from others (American Psychiatric Association, 2013), which is considered the foundation of social avoidance and has been conceptualized as hypermentalizing–essentially excessive Theory of Mind (Sharp and Vanwoerden, 2015). In social anxiety, this would present as an over-tendency to assume that others’ intentions are toward negative evaluation of them. Because this is a clear other-mentalizing error, it was reasonable to expect that other-mentalizing would moderate social impairment. Second, previous evidence suggests a differential association between self- vs. other-mentalizing, and self- vs. other-function (Ballespí et al., 2021). As such, a possible explanation to our unexpected result is that other-mentalizing may intervene in how social anxiety is developed, as an endophenotypical mechanism involved in its *appearance* (Tibi-Elhanany, 2011), but not necessarily in moderating the consequences of social anxiety once present.

### Self-Mentalization: Moderator of Social Functioning

While it is logical that self-mentalizing moderates self-functioning, the finding that self-mentalizing moderated impairment in social functioning requires some reflection. Viewing the development of social anxiety chronologically, it is possible that hypermentalizing leads to social anxiety, but the mechanism through which social anxiety impairs social functioning is in fact moderated by self-mentalizing, precisely because it is directly associated with emotional regulation (Fonagy et al., 2005). In other words, once social anxiety is present, it is less debilitating if those who experience social anxiety are aware of (and therefore more able to cope with) their experience, compared to those with less awareness and regulation, who may become inundated by their incomprehensible feelings. From this point of view, the finding that self- but not other-mentalizing moderates all evaluated functional consequences of social anxiety supports well-established evidence that social anxiety is an internalizing (self) problem (American Psychiatric Association, 2013), and is consistent with previous evidence that supports insight as an active ingredient promoting mental health (Stefan and Cheie, 2020). This suggests that the extent to which people with social anxiety are aware of and understand their socially anxious experiences is involved in their impairment, for both self- and social-function.

### Subdimensions of Self-Mentalizing: Attention vs. Clarity

Closer analysis of how self-mentalizing moderates this impairment showed differences regarding the two subdimensions of self-mentalizing, attention and clarity. While our hypothesis that the association between social anxiety and impairment would be more strongly moderated by clarity than attention was supported for social functioning, it was in fact attention to emotions that moderated the effect of social anxiety symptoms on self-functioning.

Interestingly, clarity does not simply moderate “more” than attention, but was the only significant moderator of social functioning, while the opposite finding was found for self-functioning, whereby attention moderated but clarity did not. This demonstrates that two different dimensions (attention and clarity) provide opposite moderation (strengthening and buffering, respectively) regarding two different domains of functioning (self- vs. social- functioning).

The incomplete accordance with our hypothesis led us to wonder why emotional clarity decreases social functioning impairment, while this does not occur regarding self-function. The fact that self-mentalization buffers negative effects on social functioning is consistent with the view of mentalizing as a resilience factor (Stein, 2006; Fonagy and Campbell, 2017). More specifically, this is aligned with evidence that supports emotional awareness and insight as an adaptive coping mechanism for emotional distress (Troy and Mauss, 2011; Subic-Wrana et al., 2014), an ability consistent with–or possibly necessary for–good social functioning (Sendzik et al., 2017).

In fact, emotional dysregulation in social anxiety involves attentional biases to the physiological signs of anxiety, which the individual expects and fears are perceived and negatively judged by others. Results suggest that clarity about this process buffers the impairment on social functioning. Nonetheless, for the same reason one could question why attention to emotions does not exacerbate the impairment on social functioning as it seems to with indicators of self-function.

Literature suggests that excessive attention to one’s own emotional reactions, particularly if this attention is not followed by emotional clarity, tends to exacerbate rather than buffer this reaction (Gross, 2002; Gross and John, 2003). This assertion is consistent with classic etiopathogenic models of social anxiety (Wong et al., 2014), where attention to–and therefore excessive awareness of–the physiological reaction of anxiety is expected to aggravate emotional dysregulation. However, once more, this places the role of attention in the *development* of the anxiety reaction, but not moderating the association between already present social anxiety and its functional consequences. This explanation, however, is logical for social functioning where results show that attention does not moderate the association, but not for the indicators of self-function, where attention (not clarity) is involved.

### Self-Functioning: Why Doesn’t Clarity Moderate?

While the expected outcome was revealed for attention on self-function in the present research (i.e., it worsens it), the fact that emotional clarity did not attenuate the association between social anxiety and self-functioning is an intriguing result. One possible explanation is that self-functioning impairment (i.e., in this case, decreasing self-esteem and increasing depression) appears as social anxiety tends to increase, signifying frequent and intense fear that causes individuals to avoid social interaction, thereby depriving them of the support and rewards that social relationships offer. This, in turn, would exacerbate symptoms by robbing individuals of the protective effects of social interaction (Aderka et al., 2012; Aune et al., 2021). Since attention to one’s own anxiety reaction strengthens the process that causes said social avoidance (Jakymin and Harris, 2012), and clarity is obstructed under excessive emotional arousal (Luyten et al., 2012), this could justify the idea that impairment in self-function, which is associated with consistently high rates of social anxiety, could be worsened by the same excessive attention to emotions that contribute to increasing social anxiety and impeding the protective effect of clarity in the first place. Yet another possible explanation is that attention and clarity should not be analyzed separately because they are interdependent dimensions of the same process. Therefore, how they interact with each other and moderate the relationship, could shed light on this result. Given that a 3-way interaction would not be interpretable because attention and clarity moderate in different direction, the influence of their imbalances was tested by probing moderation at low, average and high levels of both moderating variables, according to Hayes (2017).

### Impairment Varies According to Imbalances Between Attention and Clarity

When evaluating moderation at different levels of both moderators, or put simply, analyzing the conditional effect of social anxiety on impairment at different values of attention combined with different values of clarity, the analysis reveals a very consistent result within the current study and with those previously reported in the literature: the higher one’s emotional clarity and the lower one’s emotional attention is, the better the outcome. In fact, this is a combination of the two factors found to foster mental health when analyzed separately: high clarity and low attention.

In the case of social functioning, where clarity was a clear moderator that buffered impairment experienced with social anxiety symptoms, the values of the effect of social anxiety on the indicators (social competence and sociometric status) diminish in a near-linear fashion (Table 4) as clarity increases and attention decreases. Beyond the exception commented above, this result is incredibly consistent. The extreme polarities between attention and clarity show extreme changes in the effect of social anxiety regarding all outcomes. Accordingly, high attention and low clarity constitute the worst combination in all cases, and therefore the most impairment (i.e., the highest strengthening of the association between social anxiety and impairment on all four outcome variables), while the opposite, low attention and high clarity, shows the highest buffering effect. This finding extends previous evidence that high attention with low clarity is associated with mental health detriments (Gross, 2002; Gross and John, 2003; Boden and Thompson, 2017). The second finding, however, is not as well-supported by previous research, and introduces an important research question: is the positive imbalance of self-mentalizing subdimensions (low attention and high clarity) better than balanced high self-awareness (high attention and clarity) in terms of mental health? According to evidence supporting insight as a factor promoting mental health (David, 2004; Jennissen et al., 2018), it seems that high level of the both attention and clarity should provide better emotional awareness and yield the most protective effect. However, in terms of what recent literature points to regarding attention and clarity, where clarity consistently appears as the active ingredient and the negative imbalance (high attention and low clarity) as the most impairing combination (Boden and Thompson, 2017), it seems that the opposite, that is, low level of attention (which seems to be harmful) and high level of clarity (which seems to be beneficial) should reasonably be most protective for mental health outcomes.

Moreover, the debate as to whether explicit (high attention and high clarity) or implicit mentalizing (low attention but high clarity) is more advantageous for mental health naturally presents itself given this result. The current results suggest that implicit mentalizing is clearly better in terms of moderating the impairment of symptoms on functioning, as clarity with low attention does not demonstrate significantly worse effects of social anxiety on the four indicators of impairment, while clarity with high attention does (Table 4 and Figures 2–5). This is an interesting and novel finding.

On one hand, explicit mentalizing is encouraged by mentalization-based treatments (MBT) (Bateman and Fonagy, 2004) to repair mentalizing errors that appear when emotional arousal switches off explicit mentalizing and pre- mentalizing modes appear (Fonagy and Allison, 2014; Luyten and Fonagy, 2015). Pre-mentalizing modes are automatic and therefore implicit forms of “failed mentalizing.” This suggests that explicit or “full mentalizing” could be more advantageous.

However, despite the importance of explicit mentalizing, automatic processes also denote proficiency or expertise, precisely because automatization reduces resource load and allows one to utilize them for other cognitive processes (Van Merrienboer and Sweller, 2005). In light of current results, it is possible that this “low-flying” mentalizing, which seems to occur with low attention, is a more sophisticated form of mentalization which yields emotional clarity while releasing attentional resources to attend to processes outside of the self, such as those that take place in the social world. Future studies should further examine these combinations to better understand which is best for mental health resilience.

### Strengths and Limitations

A primary benefit of the present research is its novelty; to our knowledge, no research before has evaluated the moderator role of self- and other- mentalizing polarities, let alone on the impairment associated with social anxiety. This study is also the first to analyze how the association between social anxiety and impairment changes in accordance with the balance or imbalance of two self-mentalizing dimensions, attention to emotions and emotional clarity. Further, in the attempt to capture and understand mental health issues earlier in their developmental course, this research focused on a non-clinical, adolescent sample. The spectrum-based perspective that this adopts allows researchers to better understand the mechanisms involved in mental health development from a dimensional perspective. Nevertheless, as a novel result, the present research should be replicated before conclusions are drawn, especially in light of the low values of *R*^2^ of the interaction. Particularly due to its cross-sectional study design, causation cannot be ascertained, and thus the directionality and timeframe of when mentalization becomes preventative in the presence and development of social anxiety remains to be clear. Finally, although well-established psychometric measures are acceptably used, especially to assess large samples, the lack of a better measure of self-mentalizing than the self-report used in this study, is also a limitation. Given the importance of self-mentalizing as a potential general resilient factor, innovation in the assessment of this higher order cognition is a hot topic and deserves attention.

## Conclusion and Clinical Implications

Overall, the current study demonstrated that self- but not other- mentalizing moderates the association between social anxiety and different measures of impairment. This highlights the significance of mentalizing self-processes when treating social anxiety symptoms in the non-clinical range of the spectrum, though this likely also extends to the clinical population. Thus, while classic multidimensional treatments include elements such social skill training or exposure to the social world, focused on interaction, the current results stress the extent to which comprehension of one’s own emotional experience is crucial to buffer the social impairments associated with social anxiety. These implications, which currently refer to the sub- and the non-clinical range of the social anxiety spectrum, could possibly be extended to the clinical range given the dimensional perspective and continuity between clinical and non-clinical social anxiety that the notion of a continuum introduces (e.g., Schneier et al., 2002; Van Os et al., 2009; Barrantes-Vidal et al., 2015; Thapar and Riglin, 2020). Further, these results showed that attention is particularly harmful when it is not paired with emotional clarity, which leads us to suggest that future research evaluate how the subdimensions of self-mentalizing interact with each other in the complex interplay between symptoms, mentalizing, function, and resilience. Given this result, therapies and strategies that encourage emotional self-consciousness (e.g., mindfulness-based interventions, acceptance and commitment therapy, MBT) may be particularly helpful to reduce the impairment in social anxiety, and possibly further still in other psychopathologic spectra.

## Data Availability Statement

The datasets presented in this study can be found in online repositories. The names of the repository/repositories and accession number(s) can be found in the article/Supplementary Material.

## Ethics Statement

The studies involving human participants were reviewed and approved by the Ethics Committee at the Universitat Autònoma de Barcelona (CEEAH 2603). Written informed consent to participate in this study was provided by the participants’ legal guardian/next of kin.

## Author Contributions

SB conceptualized and conducted research and wrote the first draft. SB and JV validated the data, contributed to data curation and visualization, defined the methodology, and performed formal analysis. SB, JN, and JV wrote the manuscript. SB, NB-V, and JV acquired funding. All authors provided resources and critically reviewed the manuscript.

## Conflict of Interest

The authors declare that the research was conducted in the absence of any commercial or financial relationships that could be construed as a potential conflict of interest.

## Publisher’s Note

All claims expressed in this article are solely those of the authors and do not necessarily represent those of their affiliated organizations, or those of the publisher, the editors and the reviewers. Any product that may be evaluated in this article, or claim that may be made by its manufacturer, is not guaranteed or endorsed by the publisher.
